# A Medical Causerie

**Published:** 1916-07-15

**Authors:** 


### A MEDICAL CAUSERIE.

The relations between a Coroner and the members of the medical profession who are concerned with the proceedings of his Court ought not to be difficult or contentious if only there is good will, courtesy, and a recognition of responsibility on both one side and the other. Happily there are many examples which illustrate the truth of this statement. Instances in the opposite direction do occur now and again, and many of these can claim strict legality in each of the rival powers. But the position is not one in which technical rights ought to be pushed to an extreme, for in such a course friction is inevitable. A readiness to apply common-sense to the situation and a little mutual helpfulness would avoid many undignified squabbles and would save much loss of temper. Medical journals are not unnaturally ready to take note of occasions where failure in these respects occurs on the side of the Coroner. But they are ready also to recognise that sometimes the fault lies elsewhere. In a recent instance a medical practitioner who had seen the patient shortly before death, and who is reported to have taken a hopeful view of the case, was given by the Coroner an opportunity of being present at the *post-mortem* examination and of attending the inquest. But he did neither the one nor the other. Strictly speaking, no doubt, he was within his right. He was neither officially directed to make the examination nor was he authoritatively summoned as a witness. Hence he was quite free to decline both the one and the other. Whether in so doing he acted in his own interest it was for himself to decide. But it can hardly be a matter for surprise that the Coroner placed a hostile interpretation on his action and that the jury censured it. Neither the one decision nor the other has any legal value. On the other hand, the practitioner was entirely within the law. The public will form its own judgment, but is more likely to adopt the extra-legal findings than the strictly legal defence. As in these columns we have sometimes had to criticise the action of a Coroner, we are the more bound to regret that on this occasion the provocation was supplied by a member of the medical profession.

---

The action brought by the medical officers of Holloway Prison against the Women's Social and Political Union, and recently decided in the Court of King's Bench, would in ordinary times have probably excited a good deal of interest. But at present there are larger contentions on hand. The alleged libel was a statement made in the columns of the *Suffragette* to the effect that the medical officers of the prison had, when forcibly feeding certain of the women prisoners, taken the opportunity to administer to them doses of sedative and hypnotic drugs. This charge the doctors denied on oath, and the jury gave a verdict in their favour and assessed the damages at £750. It is noteworthy that the defences lodged on behalf of the Women's Press were struck out because the defendants had refused to obey an order of the Court. The paragraph in the *Suffragette* which formed the basis of the action was signed by two medical men, and one of these was joined as a defendant. But he did not appear at the trial, and it was stated on his behalf that he was engaged on important work in France.

---

Terrible threats are being uttered in reference to the fate of the medical profession at the conclusion of the war. A State Medical Service is, we are told, inevitable, and the doctors are all to be bound hand and foot by the red tape of bureaucracy. To meet the threatening danger organisation on trade-union lines is advised as the only remedy, though the mere mention of such a thing will produce a flutter in the most select of the professional dovecots. In the meantime, there seem to be certain places where the profession is well able to take care of itself. The good City of Bath has recently had some experience in this direction. Having advertised for a medical officer of health, the authorities heard, doubtless with gratification, that five candidates had entered the lists. In due course a committee was appointed to interview the applicants, and a meeting was arranged for a selected date. The meeting took place, and each of the candidates made his bow to the councillors. But each in turn announced the withdrawal of his application. The explanation is found in the fact that the salary offered by the Bath City Council is below the standard fixed by the British Medical Association, and representatives of the Association "intercepted" each of the candidates on his way to the committee of selection, with the result just stated. Such a proceeding may seem undignified to those who sit in high and comfortable places, but they have had the justification of success on many occasions, and they now seem likely to add Bath to the list of their triumphs. Public bodies have often taken advantage of the readiness of doctors to undersell one another, and it looks as if now they are to lose this opportunity.

---

The disappearance of the proposal to increase the duty on motor-cars has, of course, been welcomed by the profession, and on the whole the substitution of the added tax on petrol, from which doctors are to receive a rebate of 50 per cent., will be accepted with such degree of content as is possible to taxpayers called to bear new burdens. But the proposed abolition of the reports of certifying factory surgeons is not likely to pass without protest, and widespread indignation exists at the reduction of the fee for notifying infectious diseases from 2s. 6d. to 1s. The action is characterised as a particularly shabby one when so many doctors are abroad on military duty and are unable therefore to make their voices heard in the Parliamentary lobbies. There will be a strong demand for the re-establishment of the larger fee at the close of the war.

